# Midbrain local circuits shape sound intensity codes

**DOI:** 10.3389/fncir.2013.00174

**Published:** 2013-10-30

**Authors:** Calum Alex Grimsley, Jason Tait Sanchez, Shobhana Sivaramakrishnan

**Affiliations:** Department of Anatomy and Neurobiology, Northeast Ohio Medical UniversityRootstown, OH, USA

**Keywords:** high divalents, inferior colliculus, monosynaptic, local circuits, sound intensity

## Abstract

Hierarchical processing of sensory information requires interaction at multiple levels along the peripheral to central pathway. Recent evidence suggests that interaction between driving and modulating components can shape both top down and bottom up processing of sensory information. Here we show that a component inherited from extrinsic sources combines with local components to code sound intensity. By applying high concentrations of divalent cations to neurons in the nucleus of the inferior colliculus in the auditory midbrain, we show that as sound intensity increases, the source of synaptic efficacy changes from inherited inputs to local circuits. In neurons with a wide dynamic range response to intensity, inherited inputs increase firing rates at low sound intensities but saturate at mid-to-high intensities. Local circuits activate at high sound intensities and widen dynamic range by continuously increasing their output gain with intensity. Inherited inputs are necessary and sufficient to evoke tuned responses, however local circuits change peak output. Push–pull driving inhibition and excitation create net excitatory drive to intensity-variant neurons and tune neurons to intensity. Our results reveal that dynamic range and tuning re-emerge in the auditory midbrain through local circuits that are themselves variable or tuned.

## INTRODUCTION

Sensory systems use distinct coding strategies to represent complex stimuli. Information contained within the intensity of a sensory stimulus, for example, is coded in different ways to extract multiple features of the input. Intensity-variant codes provide information about the context of a sensory stimulus, such as previous history or regional interaction (Albright and Stoner, 2002; Bartlett and Wang, 2005). Intensity tuning allows object recognition (Riesenhuber and Poggio, 1999; Barbour and Wang, 2003b; Freiwald and Tsao, 2010) and preserves input sensitivity (Watkins and Barbour, 2008) and selectivity for communication signals (Rauschecker et al., 1995).

Variant and tuned intensity codes are found at multiple central levels of the auditory system (Sivaramakrishnan et al., 2004; Billimoria et al., 2008; Sadagopan and Wang, 2008; Barbour, 2011) and lie at the extremes of the dynamic range spectrum of sound intensity. Monotonically increasing firing rates that code almost the whole ~110 dB normal hearing range and peaked, non-monotonic functions tuned to a very small range of sound intensities, both optimize intensity information (Rees and Palmer, 1988; Davis et al., 2003; Polley et al., 2004; Watkins and Barbour, 2008). From the standpoint of a population code, the dynamic range continuum allows for plasticity in information transfer about sound level. Level variance changes to level tuning, for example, following conditioning (Polley et al., 2004) or negative gain control (Sivaramakrishnan et al., 2004), and level codes and receptive fields adapt to changing sound stimuli, shifting their operating points toward preferred sound levels (Kvale and Schreiner, 2004; Dean et al., 2005).

Firing rate codes of stimulus intensity require extensive central transformation to become efficient (Bolzon et al., 2009; Arnal and Giraud, 2012; Zelano and Gottfried, 2012). In this respect, a hierarchical approach is especially critical in the plasticity of sound intensity coding. Responses to simple tones and noises depend on intensity in highly non-linear ways in the mammalian auditory brainstem and midbrain (Sachs and Young, 1979; Young and Voigt, 1982; Nelken et al., 1997; Escabi et al., 2003) while in the cortex linearity or non-linearity appears more obviously dependent on stimulus pattern (Phillips and Hall, 1987; Phillips et al., 1994; Barbour and Wang, 2003a).

At the level of single neurons, intensity codes are complex functions of synaptic input strength and postsynaptic gain control mechanisms (Cardin et al., 2008; Murayama et al., 2009; Murphy and Miller, 2009). The dynamic range of a neuron (the range of intensities over which firing rate increases before saturating) widens with convergence of excitatory inputs. However, regulation of excitatory strength at high intensities, primarily through feedback inhibition or synaptic depression (Abbott et al., 1997; Olsen and Wilson, 2008; Pouille et al., 2009), is necessary to prevent coding ambiguity caused by premature saturation of the firing rate (Sivaramakrishnan et al., 2004).

One way to determine the optimum convergence pattern that would allow level-variant and tuned responses to sound intensity is to identify how central auditory neurons decode their input sources during prescribed changes in sound level. The convergence that results from the recruitment of auditory afferent inputs with sound pressure level (Sachs and Abbas, 1974) is inadequate to cover the ~110 dB range of normal hearing (Spirou et al., 1999; Young and Sachs, 2008), implying that ascending input alone is insufficient to generate a monotonically increasing firing rate over the whole intensity range. In the midbrain nucleus of the inferior colliculus (IC), afferent lemniscal activation in brain slices recruits local circuits that prolong synaptic responses (Sivaramakrishnan et al., 2013). At high levels of afferent recruitment, synaptic potentials have prolonged plateau depolarizations that increase the duration and rate of firing (Sivaramakrishnan et al., 2004; Sivaramakrishnan and Oliver, 2006). This suggests that afferent recruitment would increase the contribution of local circuits to sound intensity coding.

We hypothesized that a basic pattern of input convergence from two sources, extrinsic monosynaptic and local circuitry, would retain invariant aspects of the level code yet allow for stimulus-dependent compression or expansion of excitation. To examine local effects on sound intensity codes, we isolated extrinsic inputs from local sources in the IC. The central nucleus of the IC receives massive input convergence from lower auditory nuclei and corticofugal projections, and local circuits connect layers of cells that receive inputs at different frequencies (Oliver et al., 1991, 1997; Chase and Young, 2005; Chandrasekaran et al., 2013). To test the role of local circuits in forming codes of sound intensity, we applied a high concentration of divalent cations (HiDi; raised Ca^2^^+^ and Mg^2^^+^ concentrations; Frankenhaeuser and Hodgkin, 1957) locally in the IC during changes in sound intensity, to isolate monosynaptic from local inputs (Sivaramakrishnan et al., 2013). We found that as sound intensity increased, the source of recruited synapses changed from monosynaptic to local. When the two synaptic pools activated in staggered regions of the intensity spectrum, they widened dynamic range. When the two synaptic pools activated at overlapping intensities they preserved tuning.

## MATERIALS AND METHODS

CBA/Ca mice were obtained from Jackson Labs, Bar Harbor, Maine, or from our in-house breeding colonies. All animal procedures were approved by the Committee for Animal Care and Use at the Northeast Ohio Medical University and conformed to the guidelines for laboratory animal care and use published by the National Institutes for Health.

Single unit recordings were made in the IC of unanesthetized 1- to 2-month-old CBA/Ca mice using methods previously described (Sivaramakrishnan et al., 2013). Data are reported from 109 cells in 32 animals. Briefly, head fixed, awake, animals were used for recordings. Surgery to attach a head pin required to fix the animal’s head was performed under isoflurane anesthesia (1.5–2.0% in oxygen; Abbott Laboratories, North Chicago, IL, USA) and a small (~0.5 mm) opening was made in the skull to expose the dorsal surface of the IC. Recordings were performed on awake animals after a lapse of at least 1 day following surgical attachment of the head pin.

For recordings, the head was fixed in a stereotaxic apparatus at an angle of 20° to the horizontal. Single unit recordings were made with a glass pipette filled with normal artificial cerebrospinal fluid (ACSF; in mM): 130 NaCl, 3 KCl, 2 CaCl_2_, 1.3 MgSO_4_, 1 NaH_2_PO_4_, 26 NaHCO_3_; pH 7.35, or 1 M NaCl (15–20 MΩ). The recording electrode was glued to a five-barrel multi-pipette system (Havey and Caspary, 1980). One pipette of the multi-barrel was filled with ACSF containing 2.5× the normal concentration of divalent cations (2.5 HiDi; in mM): 125.5 NaCl, 3 KCl, 5 CaCl_2_, 3.2 MgCl_2_, 26 NaHCO_3_; pH 7.35). The remaining barrels contained antagonists of glycine receptors (strychnine (8 μM)) to block glycine receptors and GABA_A_ receptors (SR95531 or gabazine; 50–200 μM). HiDi and drugs were injected using pressure pulses applied to the back end of pipettes in the multi-barrel system. The five tubes of the multibarrel electrode were connected to a picospritzer (WPI) through a set of valves which allowed independent control of each barrel. A vacuum inlet connected to a second port on the picospritzer maintained a very low negative pressure (1–2 psi) on all barrels to prevent drug leakage. Injection pressures were raised above vacuum pressures, and kept low (4 6 psi, 100–500 ms) to prevent cell damage. Recovery from drug applications occurred through diffusive loss of the drug, or by application of normal ACSF or HiDi through another barrel. Chemicals were obtained from Sigma/Aldrich.

### ACOUSTIC STIMULATION

Sound was delivered through a speaker placed 10 cm in front of the animal at an angle of 15° to the midline, contralateral to the IC from which recordings were made. Acoustic stimuli were digitally synthesized and downloaded onto a digital signal processing card (AP2 multi-processor DSP card; Tucker–Davis Technologies, Alachua, FL, USA), converted to analog signals at a sampling rate of 500 kHz (model DA3-2; Tucker–Davis Technologies), filtered (model FT6-2; Tucker–Davis Technologies), attenuated (model PA4; Tucker–Davis Technologies), summed (model SM3; Tucker–Davis Technologies), amplified (model HCA-800II; Parasound, San Francisco, CA, USA), and sent to a loudspeaker (Infinity EMIT-B; Harmon International Industries, Woodbury, NY. USA). The output of the acoustic system was calibrated over a frequency range of 10–120 kHz using a condenser microphone (model 4135; Brüel and Kjaer, Nærum, Denmark) placed in a position normally occupied by the animal’s head.

#### Data acquisition and analysis

Custom software (Batlab; Dr. D. Gans, Northeast Ohio Medical University) was used to generate tone bursts and acquire data. Prior to carrying out single unit isolation, we used search stimuli consisting of tones, wide-band, and narrow band noise bursts separated by 30–60 ms. Well-isolated single units had stable spike amplitudes and shapes, and a signal-to-noise ratio >5:1. After a single unit was isolated, its characteristic frequency (CF) was determined. The CF was defined as the frequency at which the lowest sound pressure level consistently elicited stimulus-locked action potentials. We constructed tuning curves by varying frequencies in 1 kHz intervals over a frequency range that spanned the low and high cut-off points for responses at the sound level used to identify the CF. In several cells, tuning curves were also constructed over a 4–60 kHz range.

### CONSTRUCTION AND ANALYSIS OF RATE-INTENSITY FUNCTIONS

Sound pressure level was increased systematically from 0 to 96 dB SPL in 5 or 10 dB increments at 1 per second to prevent non-linearities in firing rate due to possible synaptic plasticity (Sivaramakrishnan et al., 2004), or peripheral non-linearities, which, for this study, might have complicated interpretation of the intensity-dependent activation of monosynaptic and polysynaptic inputs. Tone onset was delayed for 300 ms following the onset of recording and background rates were averaged during the 300 ms prior to the tone. Background rates were first examined for changes with sound intensity, and cells in which background firing rates changed were not included in the analysis. Background rates were subtracted from all Rate-intensity functions (RIFs). Lack of background subtraction did not alter the results.

Response onset was determined from the asymptote of first spike latency plots. RIFs were constructed by averaging firing rates over the maximum response duration, measured from the response onset. RIFs were generated with 12 repetitions at each sound intensity. Firing rates were first averaged across the 12 sweeps at each intensity, and SD determined. SD values and *t*-tests (*p* < 0.05) were used to determine whether RIFs were significantly different in HiDi or drugs. For clarity, SD error bars are not illustrated. Averages determined over other time windows, such as from the beginning of the sound stimulus or from the value of the median or lowest first spike latency, did not significantly alter the values of spike frequencies in this study. When comparing RIFs in different conditions, the maximum response duration was obtained from the group.

Rate-intensity functions were categorized as monotonic (with wide or narrow dynamic range), non-monotonic, or saturating, based on their firing rates at high sound levels (Sivaramakrishnan et al., 2004). Monotonic neurons comprised 42%, saturating neurons comprised 14% and non-monotonic neurons comprised 44% of the sample. Monotonic RIFs had firing rates that continued to increase, saturated, or declined by <20% at the highest sound levels. To examine intensity-variance over a wide dynamic range, we report data from cells with dynamic ranges >60 dB. Firing rates of non-monotonic RIFs reached a peak and then declined. A spike rate drop of ≥50% was considered strongly non-monotonic. Saturating functions displayed a steeply rising monotonic increase in spike rate, which then remained constant for at least 15 dB. RIFs illustrated are averages of 3–4 RIFs obtained at steady state.

### STATISTICAL TESTS

First spike latencies were calculated as the median value across 12 stimulus presentations. An acoustic travel time of 0.3 ms and the 0.5 ms rise and fall times of the tone were subtracted. When comparing median first-spike latencies across recorded units, we report the minimum value of the median first-spike latency obtained across the sound levels tested in a RIF. Normalization of RIFs were performed for each cell and fitted with a sigmoidal function, where appropriate (*r*^2^ values are reported in figure legends).

Results are expressed as mean ± Standard Error of the Mean. Standard deviation, when used, is indicated in the text. Significance was determined using paired *t*-test or ANOVA; *p* < 0.05 was used as a criterion for significance and the Bonferroni correction factor applied. Normality was confirmed (Origin software) before using the paired *t*-test or ANOVA. Actual *p* and *F*(df1,df2) values are indicated in the text or figure legends.

## RESULTS

We recorded neuronal discharge patterns *in vivo* in the IC of head-fixed unanesthetized mice. Our aim was to examine the effects of external and within-IC local inputs in structuring the responsiveness of neurons to the range of sound intensities that span normal hearing. We isolated responses to extrinsic inputs from those evoked by local circuits by blocking polysynaptic activity locally in the IC by applying ACSF containing a raised concentration of Ca^2^^+^ and Mg^2^^+^ (high-divalents, HiDi).

Electrical activation of lemniscal inputs in IC brain slices or acoustic stimulation using tones *in vivo* evokes a HiDi-insensitive and -sensitive component. The HiDi-insensitive component is a primarily monosynaptic input with a short onset latency. It shows little jitter during repeated lemniscal activation in slices and gives rise to most first spike latencies *in vivo*. The monosynaptic component is the only component activated at very low levels of afferent recruitment. With increased recruitment of lemniscal afferents, a second, HiDi-sensitive component prolongs the synaptic response. This second synaptic component has a longer latency than the monosynaptic component and reflects the integration of multiple polysynaptic inputs. HiDi blocks responses to these local polysynaptic inputs by raising the postsynaptic threshold for firing. 76% of IC neurons receive both monosynaptic and local inputs, 6% receive only monosynaptic inputs, and 17% receive only local polysynaptic inputs. Effects of HiDi are restricted to the side of the IC from which recordings are made. Recovery from HiDi application is rapid (<4–5 min) and recordings can be made at successive depths within the same IC during a single recording session. HiDi concentrations must be titrated to an optimal value that raises postsynaptic firing threshold slightly, but does not affect single unit isolation, spike heights, or durations *in vivo.* For IC neurons, this concentration is achieved by raising Ca^2^^+^ and Mg^2^^+^ 2.5-fold (2.5 HiDi; Sivaramakrishnan et al., 2013).

### HiDi PRESERVES FREQUENCY TUNING CURVES

**FIGURE 1:**
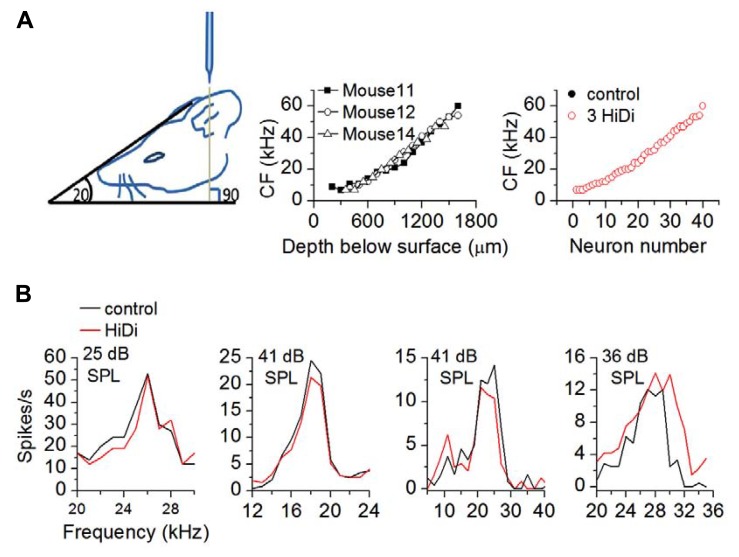
**HiDi isolates inputs that form neuronal tuning curves.** **(A)** Left: a head angle of 20° to the horizontal combined with a 90° electrode approach was optimal for accessing a wide range of characteristic frequencies. Middle: range of characteristic frequencies (CFs) in three mice. Depths were measured from the brain surface (the zero point on the abscissa). Acoustically driven responses were not observed in the first ~250 μm spanning the external and dorsal cortices. Right: sample of 40 neurons showing overlap of CF in control and HiDi (*p* = 1). The black and red circles overlap exactly. **(B)** Tuning curves are unaffected by 2.5 HiDi. Four cells are shown. Thresholds are indicated in each panel. From left to right: ANOVA: cell 1: 21–29 kHz; *p* = 0.41; cell 2: 12–22 kHz; *p* = 0.52; cell 3: 6–32 kHz; *p* = 0.37; cell 4: 20–34 kHz; *p* = 0.31.

To measure the effects of HiDi on RIFs, we measured firing rates in response to tones before and after HiDi application. Because we constructed RIFs using tones at the neuron’s CF, we first examined the effects of HiDi on CF. Recordings were made from neurons with CFs between 4 and 64 kHz, which spanned the range of CFs we were able to obtain in the IC (Egorova et al., 2006). Responses at CF were unaffected by HiDi (109 cells analyzed; *p* = 1). Frequency tuning curves were also unaffected (**Figure 1**). The different frequencies in each tuning curve overlapped (ANOVA; *p* > 0.5; *n* = 32 cells) and half-widths of tuning curves were not significantly different (*t*_63_ = 0.44; *p* = 0.66; 32 cells measured). HiDi therefore appeared to isolate CF and off-CF inputs that created IC tuning curves, suggesting that inputs at- and off-CF that form a neuron’s tuning curve comprise a group of external monosynaptic inputs to the IC.

### EFFECTS OF HiDi ON RATE-INTENSITY FUNCTIONS

We focused on two issues. First, we asked whether a wide dynamic range of sound intensity was inherited from ascending inputs or re-emerged in the IC. The mismatch between narrow dynamic range peripheral responses to pure tones and wider dynamic range responses in central neurons could conceivably occur through a smooth “stitching” (Barbour, 2011) of multiple sources that arise from the activation of the predominantly narrow dynamic range (~35 dB) peripheral excitatory inputs in different regions of the ~100 dB intensity spectrum. To test this, we used HiDi to separately examine the monosynaptic and local contribution to RIFs in neurons with dynamic ranges ≥60 dB. Second, we asked whether strong tuning to intensity (≥50% drop in firing after the peak) was inherited from extrinsic sources or formed in the IC.

Rate-intensity functions were constructed with 100 ms pure tones separated by 1 s to prevent adaptive effects on firing caused by high tone repetition rates (Sivaramakrishnan et al., 2004). HiDi was then applied with pressure pulses for several minutes through one barrel of a multi-barrel electrode (**Figure 1**) and RIFs constructed again. The RIF that remained in HiDi was due to the monosynaptic input and associated postsynaptic integration (RIF_M_). The difference between the firing rates before and after HiDi would arise from local inputs. The effect of local inputs was measured as a change in gain, GAIN_L_. GAIN_L_ was derived from the ratio of the control RIF to RIF_M_ and represents the multiplicative effect of the local circuit on output firing rate.

### LOCAL CIRCUITS WIDEN DYNAMIC RANGE

**FIGURE 2:**
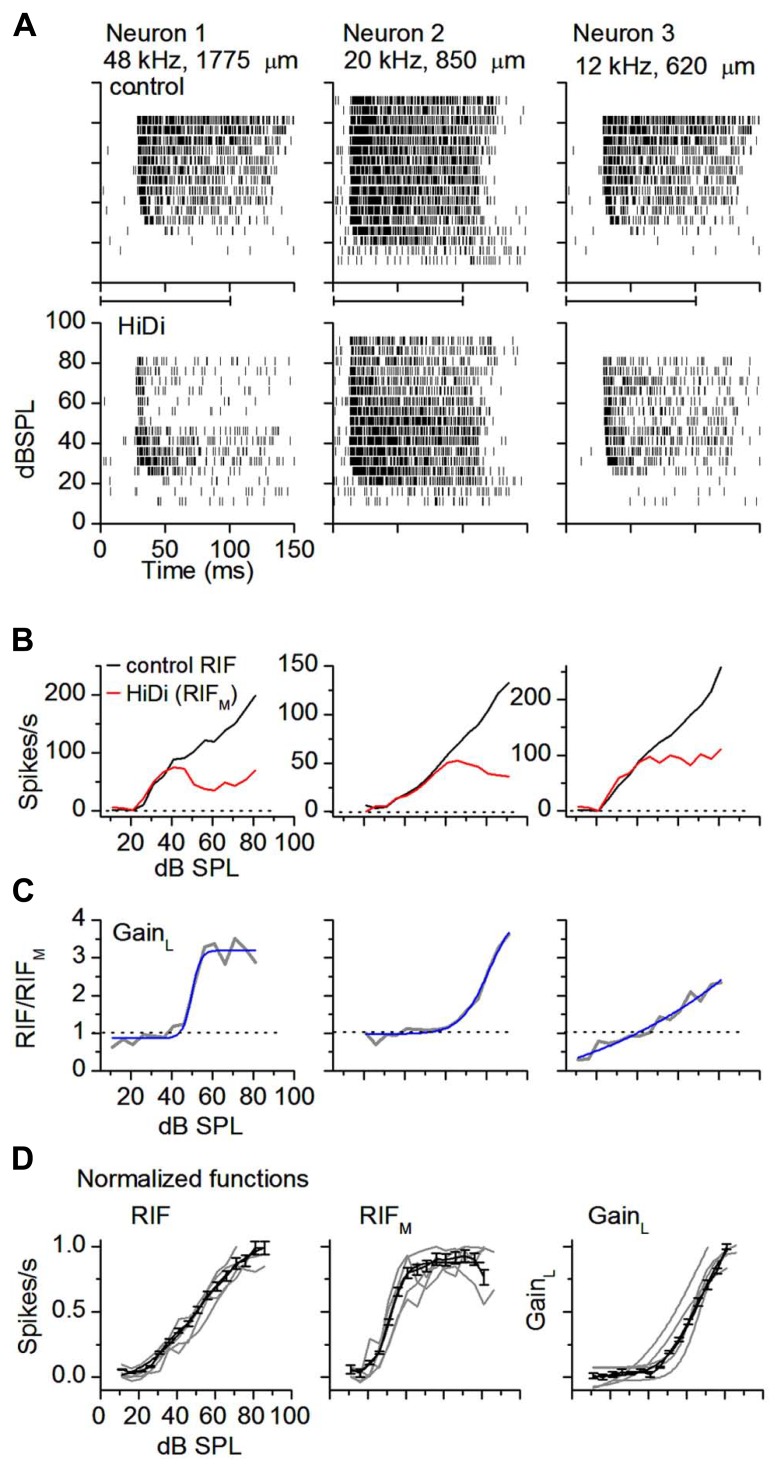
**External inputs and local circuits combine to widen dynamic range.** **(A)** HiDi decreases firing rate at mid- to high sound intensities. Spike rasters in three monotonic neurons in control (top panels) and in 2.5 HiDi (bottom panels). CFs and recording depths are indicated. Horizontal bar at abscissa: tone duration. **(B)** Rate-intensity functions show a wide dynamic range, intensity-variant, response (RIF) for each of the cells in **(A)**. HiDi decreases the dynamic range, and the RIF in HiDi, due to the monosynaptic input (RIF_M_), is saturating or slightly non-monotonic at higher intensities. Firing rates are averaged over 12 presentations of the tone at each sound pressure level. **(C)** The gain of the local circuit, Gain_L_, is the ratio of firing rates in control and HiDi. Gain_L_ = RIF/RIF_M_, increases with sound intensity. Gain_L_ is plotted as a function of sound intensity for each of the neurons in **(B)**. A gain <1 in the right panel is due to a slightly higher firing rate in HiDi at low intensities (~six spikes/s at 11 and 16 dB SPL). Sigmoidal fits. *r*^2^ from left to right: 0.95956, 0.98407, and 0.94814. **(D)** Normalized RIFs in control and HiDi and normalized Gain_L_ for five neurons with control dynamic ranges ≥60 dB (gray lines). Black lines: population average of normalized RIFs and Gain_L_. 33 cells. Mean and s.e.m. Sigmoidal fits: RIF, *r*^2^ = 0.9942; RIF_M_, *r*^2^ = 0.9846; Gain_L_, *r*^2^ = 0.9933.

In neurons with wide dynamic range responses to sound intensity >60 dB (*n* = 39), firing rates decreased in HiDi. This decrease did not depend on the neuron’s CF. Spike rasters showed clear break-points at mid-sound levels (50 ± 16 dB SPL; *n* = 15) followed by a second wave of less intense firing (**Figure 2A**, neuron 1), or a more gradual spike loss toward high intensities (**Figure 2A**, neurons 2, 3; *n* = 24). In neurons with a sustained response to the 100 ms tones we used, responses in HiDi occurred throughout the tone, thus the monosynaptic input continued to provide excitation during the tone.

If the reduced spike rate was due to a threshold increase in HiDi, then spike rates should have been preferentially reduced at low sound intensities, when excitatory input is presumably low. Firing rates at low sound intensities, however, overlapped before and after HiDi application (20–40 dB above threshold; ANOVA, *p* = 0.57; *n* = 28 cells analyzed). Because we did not find evidence of non-linearities in postsynaptic spike characteristics *in vivo* (Sivaramakrishnan et al., 2013) we assume that the RIF in HiDi was due to the monosynaptic input and associated postsynaptic integration. The monosynaptcally driven RIF (RIF_M_) had two segments. At 20–40 dB above threshold, RIF_M_ overlapped the output RIF (20–40 dB SPL; *p* = 0.57). At higher sound intensities, RIF_M_ deviated from the output RIF (50–70 dB SPL; *p* = 0.0002). In individual neurons, RIF_M_ saturated (*n* = 12), decreased slightly (*n* = 16), or was shallowly monotonic (*n* = 11; **Figure 2B**). The average trajectory shift of RIF_M_ from the output RIF occurred at 38 ± 6 dB above threshold (*n* = 39). Because neurons were able to continue to increase their firing rates beyond this sound intensity to generate the output RIF, the saturation of RIF_M_ did not arise through postsynaptic block.

The gain exerted by the local circuit, Gain_L_, is the ratio RIF/RIF_M_ (**Figure 2C**). Gain_L_ was variable. From a baseline gain of 1, it began its increase at intensities corresponding to the deviation of RIF_M_ from the output RIF (*t*_77_ = 0.34; *p* = 0.74), and then continued to increase with intensity. In 39 neurons with dynamic ranges >60 dB, the average maximum gain was 3.6 ± 1.2 at 90 dB SPL, an increase of 2.6 over its unitary gain at low intensities. The local circuit therefore multiplied neuronal output. Because the multiplicative factor itself increased with intensity, local circuits must be dynamically regulated by a changing sound intensity.

In the population of wide dynamic range (>60 dB) neurons, the output RIF, RIF_M_, and Gain_L_, were sigmoidal (*r*^2^ > 0.98; **Figure 2D**). RIF_M_ activated at the same low threshold as RIF (19.2 ± 0.9; 18.6 ± 1.1 dB SPL; *t*_77_ = 0.78; *p* = 0.44), and saturated at 48 ± 7 dB, 36 dB lower than the saturation of RIF (84 ± 8 dB SPL). The local circuit activated at mid-sound intensities (46.1 ± 9 dB) and, as an average in the population, did not saturate strongly within the range of intensities tested. Dynamic ranges of the output RIF, and the monosynaptic and local circuit components were significantly different (73.8 ± 10.7; 32.6 ± 6.3; 53.4 ± 8.4; *F*_2,96_ = 6.47; *p* < 0.002; *n* = 33 cells). The dynamic range of the output RIF was ~13 dB narrower than the combined dynamic ranges of RIF_M_ and the local circuit, and was likely due to increased K^+^ conductances (Sivaramakrishnan and Oliver, 2006). Thus monosynaptic inputs to the IC and local circuits combined to widen dynamic range.

### LOCAL CIRCUITS PRESERVE INTENSITY-TUNING

**FIGURE 3:**
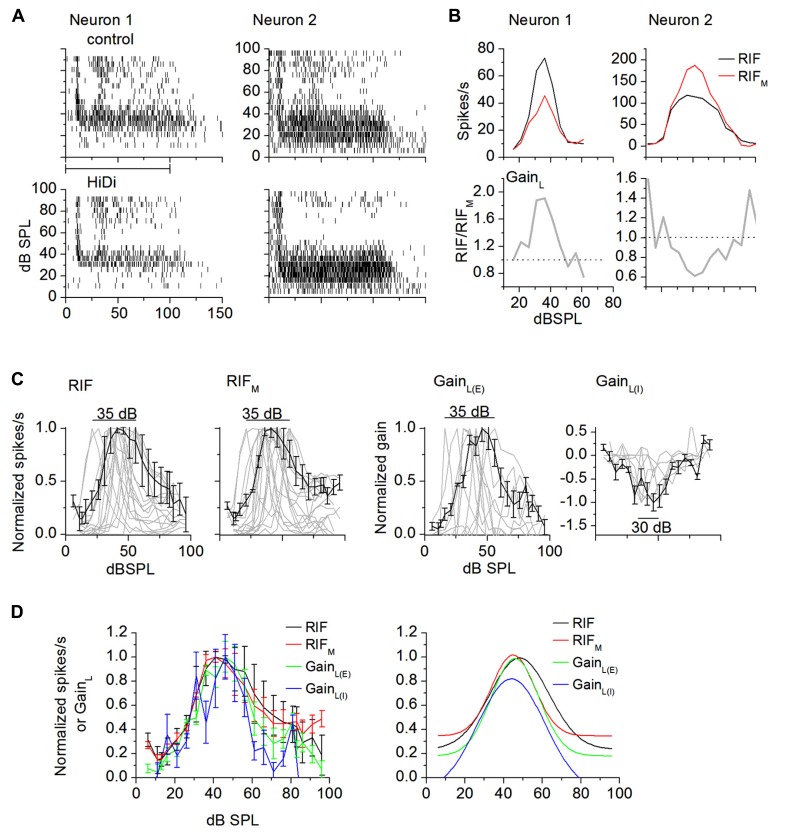
**External and local influences on intensity-tuning.** **(A)** Spike rasters of two intensity-tuned neurons (neuron 1, neuron 2) in control and HiDi. HiDi decreases peak firing rate in neuron 1 and increases it in neuron 2. **(B)** RIFs for each of the neurons in **(A)**. Neuron 1: RIF_M_ and Gain_L_ are both excitatory. Top: RIF_M_ is tuned to the same range of intensities as the output RIF. Bottom: Gain_L_, the ratio RIF/RIF_M_, is also tuned to the same intensities as the output RIF. In this neuron, the local circuit supplies a gain of 1.9 at peak tuned intensities. Dotted line: gain of 1 implies no net effect of the local circuit. Neuron 2: RIF_M_ is excitatory, Gain_L_ is inhibitory. Both RIF_M_ and Gain_L_ are tuned to the same intensity range as the output RIF. The local circuit exerts a negative gain on firing rate. **(C)** Distribution of RIFs in intensity-tuned neurons (gray lines). Normalized data. Number of cells illustrated: RIF: 16; RIF_M_: 19; Gain_L(E)_: 9; Gain_L(I)_: 8. Peaks for the output RIF, RIF_M_, and Gain_L(E)_ are distributed over a 35 dB range and, for Gain_L(I)_, over 25 dB in the population. Black lines: population averages. Mean and SD. **(D)** Left: average normalized RIFs. Mean and SD. Number of cells: RIF: 16; RIF_M_: 19; Gain_L(E)_: 9; Gain_L(I)_: 8. Gain_L(I)_ curves are normalized to the minima. Right: Gaussian fits. *r*^2^ > 0.8793 for all curves.

Sound intensity tuning is a narrow dynamic range response (Barbour, 2011), and is highly sensitive to synaptic balance (Wehr and Zador, 2003; Sivaramakrishnan et al., 2004; Wu et al., 2006; Tan et al., 2007). In neurons that were strongly tuned to intensity (>50% reduction in firing rate at high sound intensities; Sivaramakrishnan et al., 2004; Barbour, 2011), HiDi changed firing rates in 46/52 cells. Peak firing rates in HiDi were less than the peak of the output RIF in most cells (31/46 cells; *t*_61_ = 3.07; *p* = 0.003; **Figures 3A,B**, left panels). In other cells, peak firing rates in HiDi were more than peak RIF (15/46 cells; *t*_29_ = 3.12; *p* = 0.004; **Figures 3A,B**, right panels). The net (excitatory + inhibitory) monosynaptic input was therefore sufficient to generate intensity-tuned responses and was tuned to the same intensity range as the output RIF.

In neurons in which peak firing rates in HiDi were lower than those in control conditions, the net local input increased responsiveness around tuned intensities to produce the higher firing rates of the output RIF. The gain of this net excitatory local input, Gain_L_, increased with intensity, peaked and then decreased with further intensity increases (**Figure 3B**, bottom panel; neuron 1) with a gain of 1.66 ± 0.57 (*n* = 31). In neurons in which peak firing rates in HiDi were higher than in the control, the net local input decreased responsiveness around tuned intensities to lower the firing rates of the output RIF. Gain_L_ decreased with intensity, reached a trough, and then increased again (**Figure 3B**, bottom panel; neuron 2), with a gain of -0.42 ± 0.38 (*n* = 15). Local inhibition therefore exerts a divisive effect on the output RIF. This divisive effect increases with sound intensity, consistent with the recruitment of inhibitory local inputs and/or a larger driving force on inhibitory synaptic conductances. In the excitatory and inhibitory classes of local inputs, Gain_L_ was tuned to the same range of intensities as the output RIF. The local input therefore either boosted or suppressed peak firing, but preserved the tuned region.

The intensity range over which peak firing rates were spread was inherited from monosynaptic inputs. HiDi did not change the range of intensities covered by the population of intensity-tuned peaks. Output RIF peaks were distributed narrowly, over 35 ± 5 dB (20–55 dB), as previously reported in the unanesthetized IC and auditory cortex (Sivaramakrishnan et al., 2004; Barbour, 2011, but see Sadagopan and Wang, 2008). RIF_M_ (35 ± 6 dB) and Gain_L__(E)_ and Gain_L(I)_ (excitatory and inhibitory local gain respectively; 35 ± 8dB) peaks were distributed over similar dB ranges as the output RIF (**Figure 3C**; *F*_4,204_ = 0.44; *p* = 0.78; *n* = 52). Population averages of the output RIF, the monosynaptic and local components peaked at ~41 dB SPL (**Figure 3D**; *F*_4,204_ = 1.82; *p* = 0.13; *n* = 52).

### TEMPORAL ACTIVATION OF LOCAL CIRCUITS IN INTENSITY-VARIANT AND TUNED NEURONS

The prolonged nature of polysynaptic responses to afferent lemniscal stimulation in IC brain slices (Sivaramakrishnan et al., 2013) suggested that local circuits would be preferentially activated at later times during a tone. Analysis of RIFs in distinct onset and sustained regions of the tone suggested that tone duration contributed to both dynamic range and tuning.

**FIGURE 4:**
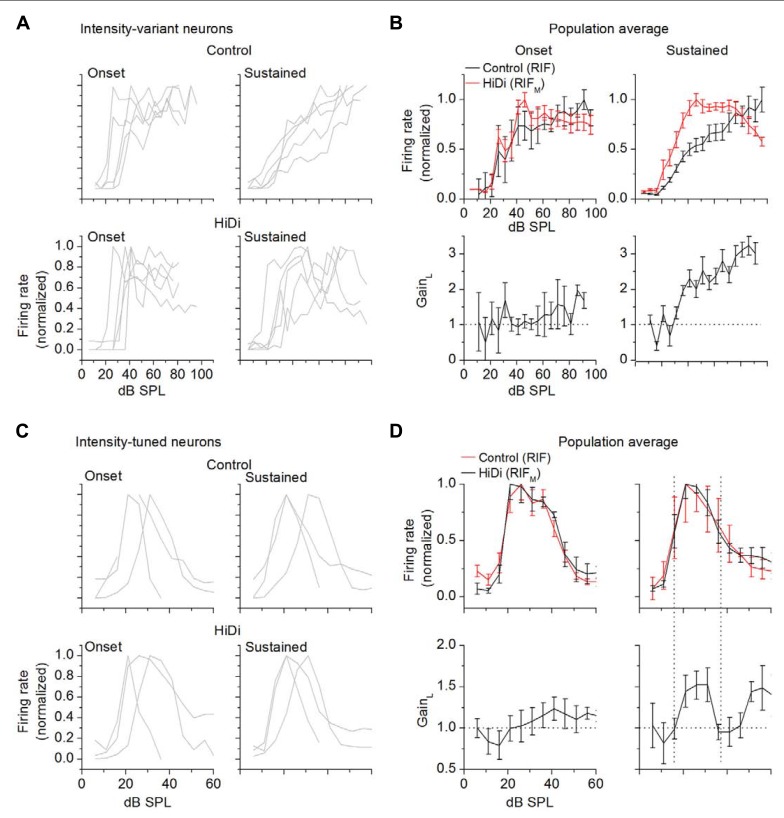
**Temporal activation of monosynaptic and local inputs.** For both intensity-variant and tuned neurons, onset responses are averaged over the first 20 ms; sustained responses are averaged between 25 and 100 ms. Response onsets are measured from the mean first spike latency. RIFs are normalized. All data are from cells that exhibited a sustained response during a 100 ms tone. **(A,B)** Intensity-variant neurons. **(A)** Onset and sustained responses in control (top) and HiDi (bottom) for five cells with dynamic ranges >60 dB. **(B)** Population averages. Twelve cells. Mean and SD. The RIF due to the monosynaptic input has a short dynamic range during both the onset and sustained portions of the response to the tone. Gain_L_ was measured at 30, 60, 70, 80 dB SPL prior to normalization of the control and HiDi RIFs and averaged across cells. Local circuit gain does not increase during the onset portion (*F*_4,44_ = 1.22; *p* = 0.31), but increases during the sustained response (*F*_4,44_ = 9.72; *p* < 10^-^^5^). **(C,D)** Intensity-tuned neurons. **(C)** Onset and sustained responses in control (top) and HiDi (bottom) for three cells with different tuning widths. **(D)** Population averages. 14 cells. Mean and SD. Since the HiDi and control functions were normalized, their peaks overlap. A slight increase in gain occurs during the onset portion of the tone (*F*_4,52_ = 2.74;* p* = 0.038). Strong local circuit activation during the sustained portion of the tone occurs during the tuned region (vertical dotted lines). Gain_L_ was measured prior to normalization of the control and HiDi RIFs and averaged across cells.

In intensity-variant neurons, RIF and RIF_M_ were both steeply saturating functions during the onset portion (the first 20 ms following response onset) of the tone. At later times (25–100 ms), the output RIF increased monotonically with a wide dynamic range, whereas RIF_M_ remained a short dynamic range, saturating function (**Figures 4A,B**). As an average in the population, in the onset and sustained portions of the tone, RIF_M_ saturated at a similar sound intensity (41 ± 6 and 43 ± 5 dB SPL, respectively). Gain_L_ remained at 1 for all intensities during the onset portion of the tone (tested at 30, 50, 70, 80 dB SPL; *F*_4,44_ = 1.22; *p* = 0.31), but increased during the sustained portion (*F*_4,44_ = 9.72; *p* < 10^-^^5^), reaching a maximum gain of ~3 by 80 dB SPL. Integration during the tone therefore appears to favor activation of local circuits. The increase in output gain (by a factor of 3 at 80 dB SPL) suggests that integration during the tone results in non-linear changes in local circuits.

In intensity-tuned neurons, the output RIF and RIF_M_ remained similarly tuned during the onset and sustained portions of the tone (**Figures 4C,D**). The monosynaptic input therefore remained consistent with integration. During the onset portion of the tone, GAIN_L_ increased slightly at higher intensities (tested at 20, 30, 40, 50 dB SPL; *F*_4,52_ = 2.74; *p* = 0.038; *n* = 14 cells), corresponding to the falling limb of the output RIF. With integration over the later part of the tone, however, GAIN_L_ was strongly tuned, with a tuned region that corresponded with that of RIF. Gain_L_ increased to 1.5 during the tuned region (difference between baseline gain and maximum gain during the tuned region; *t*_27_ = 3.19; *p* = 0.004; *n* = 14 cells). Additional changes in gain occurred during the falling limb of RIF. Between 20 and 40 dB SPL, within the tuned region, the average change in Gain_L_ was higher during the sustained portion of the tone (increase from baseline gain of 0.55 ± 0.21) compared with the onset portion (increase of 0.13 ± 0.22 from baseline gain; *t*_27_ = 2.57; *p* = 0.01; *n* = 14).

### PUSH–PULL GAIN CONTROL BY MONOSYNAPTIC INPUTS

The inability of RIF_M_ to reach the peak firing rates of the output RIF in intensity-variant and in intensity-tuned neurons where HiDi reduced peak firing rates (as in **Figures 2** and **3**) suggested a saturation of the net monosynaptic input. This saturation might reflect saturation of ascending excitation, or might be due to the strong inhibition that the IC receives from brainstem sources (Cant and Benson, 2003). Decreased excitatory input accompanied by increased inhibitory input, or vice versa, produces a push–pull gain control of neuronal output by mutual reinforcement (Ferster, 1988) and typically occurs through increased conductance of the postsynaptic membrane due to the inhibitory input (Steriade, 2001; Destexhe et al., 2003). Push–pull gain control shapes sensory receptive fields (Ferster and Miller, 2000; Hirsch and Martinez, 2006) and has been suggested to be a characteristic feature of driving inputs (Abbott and Chance, 2005).

To determine whether the saturation of RIF_M_ reflected excitatory saturation alone or included monosynaptic inhibition, we recorded firing rates first in HiDi, and then after blocking (monosynaptic) inhibition with antagonists of GABA_A_ (gabazine, Gz) and glycine (strychnine) receptors. We dissolved the antagonists in HiDi to prevent re-activation of local inputs.

**FIGURE 5:**
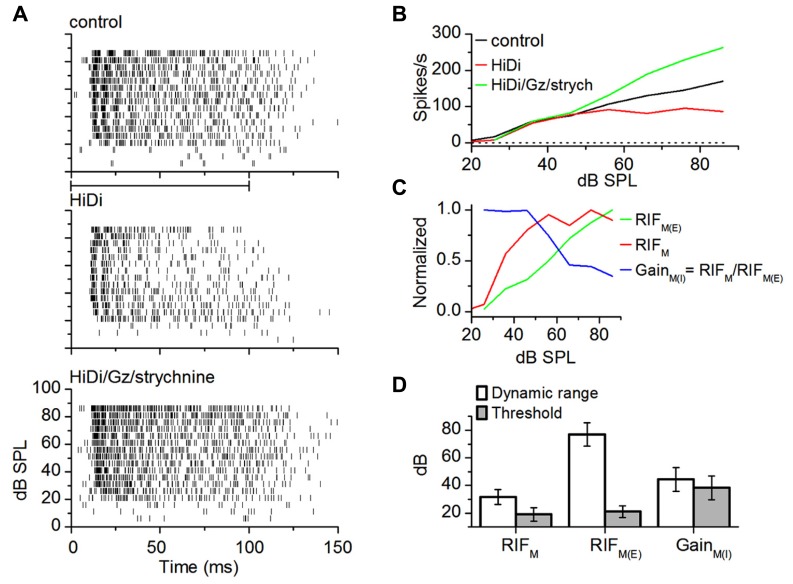
**Monosynaptic excitation and inhibition in wide dynamic range neurons.** **(A)** Spike rasters. Firing rates decrease in HiDi (middle), but increase again in the inhibitory antagonists (bottom). Gabazine (Gz, 50 μM); strychnine (8 μM). The inhibition is a monosynaptic input. **(B)** RIFs for the cell in **(A)**. The RIF in HiDi/Gz,/strychnine, due to the excitatory component of the monosynaptic input increases throughout the range of intensities, unlike the net monosynaptic input, RIF_M_, which saturates. **(C)** RIF_M_ consists of an excitatory component, RIF_M(E)_, and an inhibitory monosynaptic component which exerts a gain, Gain_M(I)_ = RIF_M_/RIF_M(E)_. Push–pull interaction between monosynaptic inhibition and excitation generates the net monosynaptic input. RIF_M(E)_/RIF_M_ slope ratios: rising limb, 2.12 ± 0.085; falling limb, 2.24 ± 0.13; **(D)** average threshold and dynamic range. 16 cells. Mean and SEM.

In neurons with wide dynamic ranges, spike rates dropped in HiDi and increased again in inhibitory antagonists (**Figure 5A**; *n* = 16 cells). The RIF in HiDi/Gz/strychnine was due to monosynaptic excitation. Monosynaptic excitation increased continuously with intensity (up to ~90 dB SPL; *n* = 16; **Figure 5B**). Because the excitatory component diverged from the net monosynaptic input (at 42 ± 12 dB above threshold; *n* = 16), the saturation of the monosynaptic input was due to inhibition. The gain of monosynaptic inhibition (net monosynaptic/excitatory component) decreased with intensity, while the excitatory component increased (**Figure 5C**). Monosynaptic excitation and inhibition thus produced push–pull gain control of total extrinsic input. This finding supports the suggestion that push–pull excitation–inhibition is a characteristic of driving inputs (Abbott and Chance, 2005).

The threshold and dynamic range of monosynaptic excitation were similar to that of the output RIF (*t*_31_ = 0.73; *p* = 0.47; *n* = 16; **Figure 5D**). The excitation–inhibition balance determined first spike latencies, which were shortened in HiDi/Gz/strychnine (HiDi/Gz/strychnine 12.65 ± 3.92 SD; control 15.34 ± 51.5 SD; *t*_31_ = 2.33; *p* = 0.01) but not in HiDi alone (*t*_31_ = 1.53; *p* = 0.13; control 15.34 ± 5.15; HiDi 14.58 ± 4.03).

**FIGURE 6:**
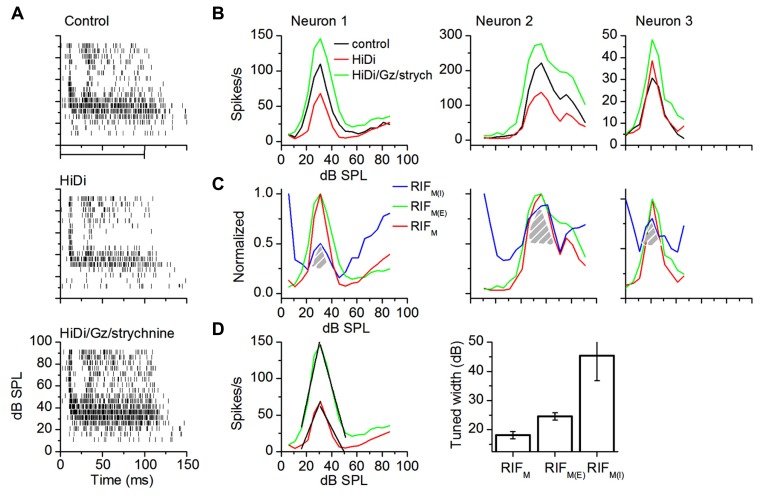
**Monosynaptic excitation and inhibition in intensity-tuned neurons.** **(A)** Spike rasters in control (top); HiDi (middle); HiDi + Gz + strychnine (bottom). **(B)** RIFs for the three neurons. Left: the cell in **(A)**; middle, right: two other cells. **(C)** Normalized RIF_M_, RIF_M(E)_, and Gain_M(I)_ for the three cells in **(B)**. Gain_M(I)_ changes the direction of its gain control with sound intensity. Hatched region: Gain_M(I)_ exhibits a “tuned” gain. **(D)** Left: linear fits (black lines) of the rising and falling limbs of RIF_M(E)_ and RIF_M_ for Neuron 1 in **(B)**. Rising limb: RIF_M_, *r*^2^ = 0.90285; slope, 4.12821 spikes/s/dB SPL; RIF_M(E)_; *r*^2^ = 0.92772; slope, 8.2253; falling limb: RIF_M_, *r*^2^ = 0.88935; slope, -3.30769; RIF_M(E)_, *r*^2^ = 0.98401; slope, -6.3333. Right: population averages of tuned widths. Tuned widths were measured at half the peak height of normalized functions. 14 cells. Mean and SEM.

In intensity-tuned neurons (*n* = 14), the excitatory component increased firing rates over the net input (**Figure 6A**). Monosynaptic excitation remained tuned and peak firing rates occurred in the same intensity range as that of the net input (**Figure 6B**, green trace; *t*_27_ = 0.23; *p* = 0.82). Monosynaptic inhibition opposed excitation in the flank regions of the input (**Figure 6C**). Inhibition decreased (by 68.6 ± 7.3%; *n* = 14) during the rising limb of excitation and returned to a baseline gain of ~1 (84.6 ± 8.82%) during the falling limb. Between the flanks, inhibitory gain was co-tuned with excitation (**Figure 6C**, shaded areas). Excitation contributed symmetrically to the total monosynaptic input (**Figure 6D**, left; rising and falling slopes were symmetrically steeper than the net input, by approximately twofold; *n* = 14). The excitatory and inhibitory components both had wider tuning widths than the net monosynaptic component, suggesting a push–pull control of tuning width by monosynaptic excitation and inhibition (**Figure 6D**, right; *n* = 14 neurons; RIF_M_, RIF_M(E):_ *t*_27_ = 3.56; *p* = 0.0014; RIF_M_, RIF_M(I)_: *t*_27_ = 3.55.516; *p* < 0.00001).

## DISCUSSION

The goal of our study was to determine the pattern of input convergence that would allow changes in sound intensity to be represented in parallel as intensity-variant and tuned codes. To characterize input pattern, we isolated synaptic inputs based on their source, inherited monosynaptic, or local, polysynaptic, while introducing sounds of different intensities. From the responses of neurons to these two synaptic compartments, we were able to predict regions of the sound intensity code that were more or less susceptible to adaptive gain control.

### MONO- AND POLYSYNAPTIC INPUTS CREATE INVARIANT AND VARIABLE CODING DOMAINS

At low sound levels, the net monosynaptic input (excitatory + inhibitory) generated the steepest part of the RIF. It carried information about threshold, dynamic range, CF, and first spike latency. The ~35 dB dynamic range and saturating RIF suggest that monosynaptic inputs are part of the pathway that includes narrow dynamic range (~35 dB) auditory afferents whose recruitment increases with sound level (Sachs and Abbas, 1974). These afferent contacts, if made through large glutamatergic terminals or dense terminal arbors (Winer, 2005; Nakamoto et al., 2013) on proximal dendrites, would have the properties of driving inputs (Sherman and Guillery, 1998). Our results suggest that these driving inputs include those that create tuning curves, which are frequency specific channels that persist through the auditory pathway (Liu et al., 2007; Kandler et al., 2009; Sumner et al., 2009). A primary role of narrow dynamic range peripheral afferents may therefore be to ensure throughput of the rate-level code through proximal monosynaptic inputs. Ascending brainstem inputs are spread over a wide area and likely drive a broad range of cells with different CFs (McAlpine et al., 1998). Driving inputs with diverse strengths interacting with different intrinsic operating ranges of IC neurons would cause dynamic changes (Hasenstaub et al., 2007) in local IC circuits, increasing or decreasing their gain with changes in sound intensity.

The sensitivity of sound intensity codes to the pattern of sound stimuli provides clues to the changing nature of synaptic inputs to central neurons during a change in intensity. Changes in tone repetition rate, addition of tonic noise, modulation of sinusoidal amplitude, and selecting stimuli for most probable sound levels alter dynamic ranges and receptive fields (Rees and Palmer, 1988; Joris et al., 2004; Nelson et al., 2007; King et al., 2011). The intensity code is therefore highly plastic, and synaptic input must adjust dynamically to allow for the invariant and mutable regions of the level code, both of which are required to interpret changes in sound level. Convergence of narrow dynamic range (~35 dB) peripheral excitatory afferents appears more conducive to retaining the invariant than variant aspects of level codes (Carlyon and Moore, 1984; Gibson et al., 1985; Spirou et al., 1999). Afferent excitation is also strong and rises steeply, which non-intuitively narrows dynamic range in central neurons by pushing target cells to their operating limits, causing premature firing rate saturation (Sivaramakrishnan et al., 2004).

Our results show that local input fine-tunes and filters intense excitation, conferring plasticity to the system. Local recruitment would favor non-linear processes involving multiple excitatory and inhibitory sub-domains of local inputs in the IC and are likely to underlie much of the spectrotemporal complexity that appears at high sound intensities (Lesica and Grothe, 2008). Extensive connections within IC frequency laminae (Wallace et al., 2012) and axonal collateralizations (Oliver et al., 1991) are likely to recruit the majority of local neurons with increasing sound intensities. Frequency representation in the IC broadens with sound intensity, and while this is generally attributed to an increased inherited input strength, our results suggest that extrinsic input saturates at mid-sound intensities, and further increase in input recruitment occurs at the local level. Local recruitment could be triggered by commissural connections that serve as a means of di- or polysynaptic input (Moore et al., 1998). Cooling of the commissure has been recently shown to preserve short latency (<20 ms) responses to acoustic input while selectively blocking longer-latency (>20 ms) responses (Orton et al., 2012). This separation of early and late components by commissural blockage is similar to the time courses of the HiDi-insensitive and sensitive components of tone-evoked responses in our study and strengthens our hypothesis that the short latency response is evoked by a direct ascending monosynaptic lemniscal contact, while the longer latency components are driven by local-circuits. Optical imaging with voltage-sensitive dyes in IC slices suggests that commissural propagation is a high-threshold pathway, evoked either by increasing excitation or by reducing inhibition in the opposite IC (Chandrasekaran et al., 2013), and might partly account for our finding that the HiDi-sensitive local circuit is a high-threshold input.

The exact complement of inputs and postsynaptic membrane properties that influence changes in firing rate would be expected to vary with the complexity of sound stimuli. Our data suggest that the local circuit activates at high intensities, contributing a high-threshold component of sound intensity codes. NMDARs activate close to the resting potential in a substantial population of IC neurons (Wu et al., 2004; Sivaramakrishnan and Oliver, 2006) and local circuit regulation of dendritic excitability involving glutamate receptors or voltage-gated channels (Yu and Salter, 1999; Ohtsuki et al., 2012; Lee et al., 2013) would be expected to provide the multiplicative gain, which we report is about 3. Thus a combination of several factors, including variations in the complexity and number of synapses involved in the circuit, intrinsic membrane conductances, and slow acting transmitter systems, likely play a role in the dynamic change in gain of the local circuit. Feedback gain enhancement by local circuits has to be balanced by the relatively short operating range of IC neurons, the majority of which go into depolarization block at membrane potentials as negative as -30 mV (Sivaramakrishnan et al., 2004). Local inputs at high sound levels could consist of mixed excitation and inhibition with a variable synaptic gain that prevents premature firing block of the postsynaptic cell.

### TUNED AND WIDE-DYNAMIC RANGE NEURONS BELONG TO STEREOTYPIC MICROCIRCUITS

Tuned and wide-dynamic range responses to sound intensity are created and maintained by distinct synaptic infrastructures. Intensity tuning itself appears to be independent of synaptic source. The restricted spread of peaks to 35 dB, which is unaffected by input source, suggests that peak distribution does not emerge in the IC. Wide-dynamic range responses, on the other hand, are composed of sub-domains of narrow dynamic range inputs with high efficacies in non-overlapping intensity regions. Our results suggest that intensity-tuned and wide dynamic range neurons belong to different IC microcircuits that either linearly integrate inherited inputs, or bypass subsets of inherited inputs to create emerging non-linearity. The hierarchical organization between extrinsic inputs and local circuits suggests a match between intensity-variance and tuning, so that a wide dynamic range response, which can be considered as a change in gain with intensity, is formed in a neuron that belongs to a circuit that itself changes its gain with intensity. An intensity-tuned neuron which inherits its tuning from the brainstem belongs to a circuit that is itself tuned to the same range of intensities as the inherited input. This type of inherited input-local circuit match seems similar to the stereotypic circuit pattern that has been suggested for the neocortex (Silberberg et al., 2002).

## Conflict of Interest Statement

The authors declare that the research was conducted in the absence of any commercial or financial relationships that could be construed as a potential conflict of interest.

## AUTHORS CONTRIBUTION

CAG, JTS, and SS collected data. CAG and SS analyzed data. SS designed the study and wrote the paper.
